# Obstructive jaundice caused by traumatic neuroma of the common bile duct: A case report and literature review

**DOI:** 10.1097/MD.0000000000044717

**Published:** 2025-09-19

**Authors:** Guanbai Cao, Tingting Zhu, Zhao Chen, Jun Wu

**Affiliations:** a Hepatobiliary Surgery, Chongqing Jiulongpo District People’s Hospital, Jiulongpo, Chongqing, China; b Department of Pain, Chongqing Jiulongpo District People’s Hospital, Jiulongpo, Chongqing, China.

**Keywords:** common bile duct, diagnosis, neuroma, traumatic, treatment

## Abstract

**Rationale:**

Obstructive jaundice caused by traumatic neuroma of the common bile duct is extremely rare and easily misdiagnosed.

**Patient concerns:**

We herein report a 66-year-old female who presented with obstructive jaundice. Magnetic resonance cholangiopancreatography showed common hepatic duct stenosis accompanied by intrahepatic bile duct dilation. Nine months ago, a laparoscopic cholecystectomy was complicated by the transection of the common bile duct, which was treated with end-to-end anastomosis and T-tube drainage. After readmission, the patient underwent an exploratory laparotomy.

**Diagnoses:**

A soft tissue mass was intraoperatively identified to cause a common hepatic duct stricture. The frozen section of the mass showed a high possibility of neuroma. Postoperative immunohistochemistry staining confirmed a neuroma of the common bile duct.

**Interventions:**

Neuroma resection and Roux-en-Y hepaticojejunostomy were performed.

**Outcomes:**

Postoperative recovery was uneventful. She was well after a postoperative 9-year follow-up.

**Lessons:**

During cholecystectomy, the cystic duct and common hepatic duct should be carefully identified. In cases where severe inflammation or fibrosis in the Calot triangle makes dissection of the cystic duct difficult, a subtotal cholecystectomy can be done. Frozen section pathology examination during surgery can help avoid unnecessary, extensive radical surgery.

## 1. Introduction

Neuroma of common bile duct is a rare condition that may result from injury or surgical trauma to common bile duct. It involves the abnormal proliferation of nerve fibers and connective tissue at the site of injury, leading to the formation of a benign, tumor-like lesion. This condition can cause symptoms such as obstructive jaundice, abdominal pain, or cholangitis due to the obstruction of bile flow. It is important to differentiate it from malignant tumor to avoid unnecessary aggressive treatments. Here, we reported a case of obstructive jaundice caused by traumatic neuroma of common bile duct and made a review of literature.

## 2. Patient information

66-year-old Chinese Han female who was initially admitted to our hospital, with a month history of symptomatic gallstones. Laparoscopic cholecystectomy was complicated by accidental transection of common bile duct, which was treated by end-to-end anastomosis and T-tube drainage. T-tube was removed at postoperative 6-month. Nine months postoperatively, she was readmitted with a month history of yellowing of skin and sclera.

## 3. Clinical findings

Upon physical examination, she had jaundice and slightly bulging abdomen, no tenderness or muscle tension. Bowed sounds were normal. All of her vital signs were also normal.

## 4. Diagnostic assessment

**Figure 1.**
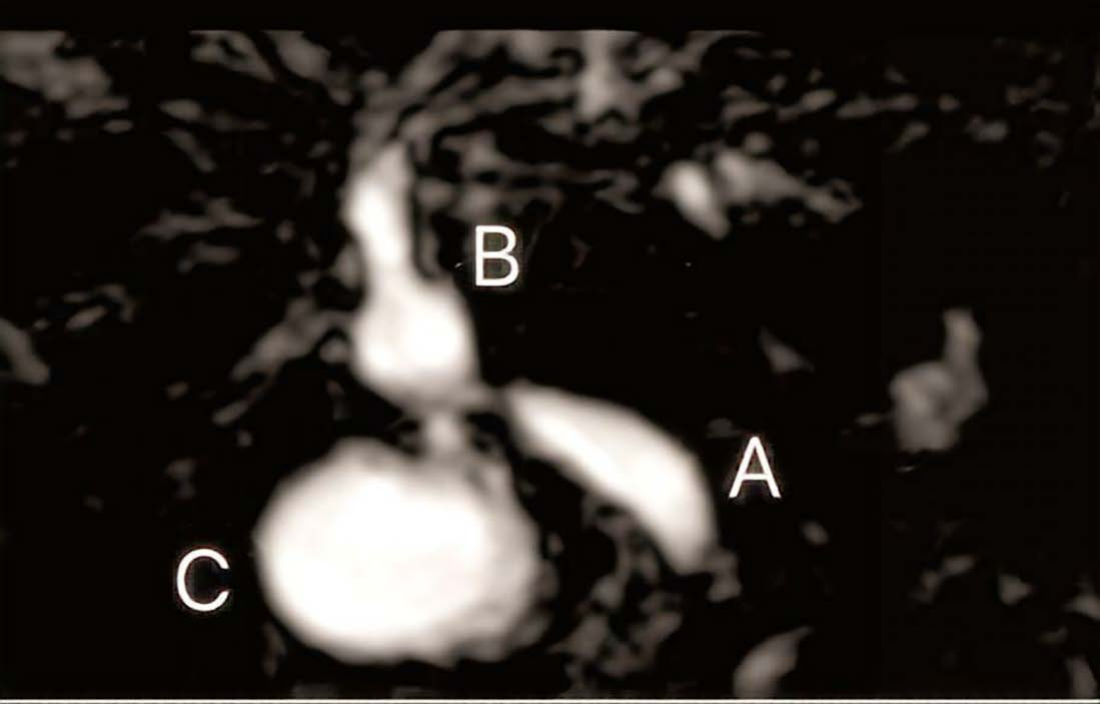
MRCP showed (A) Lower end of biliary anastomosis; (B) upper end of biliary anastomosis; (C) hydrops. MRCP = magnetic resonance cholangiopancreatography.

Laboratory investigations were notable for a raised serum total bilirubin (47.9 μmol/L; normal 4–19), direct bilirubin (39.2 μmol/L; normal 0–5), alkaline phosphatase (273 U/L; normal 40–129U/L), and gamma-glutamyltransferase (586 U/L; normal 8–61). Abdominal ultrasound showed probable presence of stones in the common hepatic duct, dilation of the common hepatic duct and intrahepatic bile ducts. Magnetic resonance cholangiopancreatography displayed common bile duct stricture with intrahepatic bile duct dilation (Fig. 1). Our provisional diagnosis was common hepatic duct stricture with intrahepatic bile duct dilation and obstructive jaundice caused by calculus of bile duct.

## 5. Therapeutic intervention

**Figure 2.**
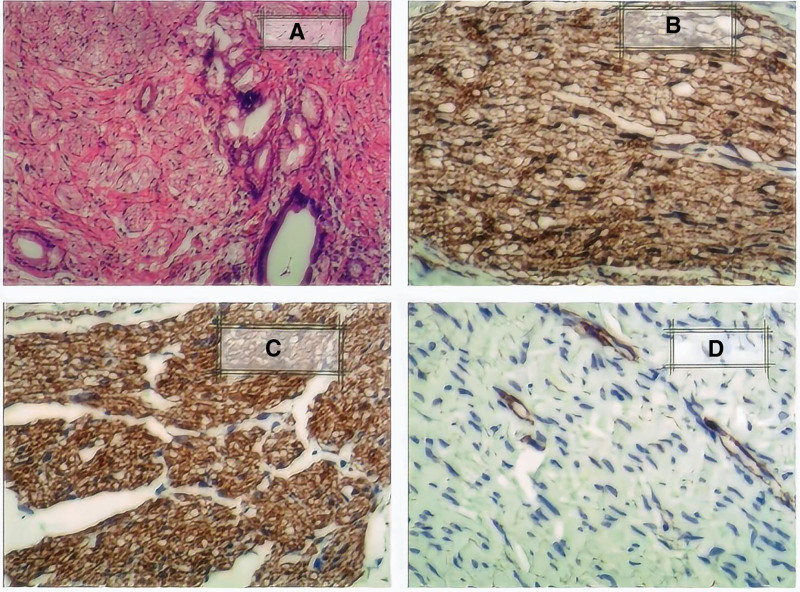
(A) Nerve fibers are seen around normal bile ducts (hematoxylin and eosin staining, 10 × 10-fold); (B) soluble in 100% saturated ammonium sulfate protein positive (immunohistochemistry staining, 10 × 20-fold); (C) neuron-specific enolase positive (immunohistochemistry staining,10 × 20-fold); (D) smooth muscle actin negative (immunohistochemistry staining, 10 × 20-fold).

The patient underwent exploratory laparotomy under general anesthesia. After dissecting adhesions, stricture at the bile duct anastomosis site caused by a 10 mm × 8 mm × 5 mm hard mass with clear boundaries was found. Common hepatic duct proximal to the anastomosis was dilated (15 mm diameter). No stricture and stones in the pancreatic segment of common bile duct. The mass was completely removed. Frozen section analysis showed benign soft tissue tumor, likely neuroma. Roux-en-Y hepaticojejunostomy was performed. Microscopic findings were nerve fibers of bile duct with Soluble in 100% saturated ammonium sulfate protein (S-100 protein) positive, neuron-specific enolase positive and smooth muscle actin negative by immunohistochemistry (IHC) staining (Fig. 2). Postoperative diagnosis was traumatic neuroma of bile duct.

## 6. Follow-up and outcomes

Postoperative recovery was uneventful. She was well after postoperative 9-year follow-up.

## 7. Discussion

Traumatic neuroma mainly occurs in the limbs and neck, rarely in the biliary tract. After common bile duct is injured, disrupted nerve fibers and fibrous tissue undergo abnormal regeneration, which may form a disorganized mass composed of nerve fibers, fibroblasts and scar tissue. This mass can compress or obstruct common bile duct and develop clinical symptoms. Traumatic neuroma of common bile duct poses a significant diagnostic challenge due to the nonspecific clinical presentation, lack of accurate imaging tool and a wide range of differential diagnoses, such as fibrosis after biliary inflammation, biliary stones, scar stricture after biliary surgery, postoperative anastomotic ischemia, iatrogenic injury, primary sclerosing cholangitis, chronic pancreatitis, cholangiocarcinoma, metastatic lymphoma, and neuroendocrine tumor. The most common cause of common bile duct stricture caused by primary tumor of extrahepatic bile ducts is invasive adenocarcinoma. Whereas benign tumors, such as adenoma, papiloma, and neuroma are much less common.

The traumatic neuroma usually manifests as hard, oval and slow-growing mass under gross appearances. The pathological features are characterized by the presence of nerve fiber, S-100 Protein positivity by IHC staining, and no evidence of malignancy. Symptoms are similar to other bile duct issues. Some patients can present with obstructive jaundice due to bile flow obstruction, abdominal pain in the right upper quadrant, fever and chills if cholangitis develops, and elevated levels of liver enzymes and bilirubin due to bile duct obstruction. A systematic review with 43 patients in 24 case reports by Lalchandani P et al show that the common risk factors for developing biliary traumatic neuroma are open cholecystectomy, liver transplantation, and uncomplicated laparoscopic cholecystectomy.^[1]^ The time from initial surgery to diagnosis of biliary traumatic neuroma ranges from 2 months to 46 years, with a median of 5 years; and in the subgroup subject to open cholecystectomy, the median time is more than 12 years.^[1]^ The interval from surgery to diagnosis in our case was 8 months.

Besides nonspecific clinical presentations, image features are also limited in narrowing down the diagnosis for traumatic neuroma of the bile duct. The imaging features of biliary traumatic neuroma are similar to that of soft tissue on ultrasonography, computed tomography and magnetic resonance imaging. Some patients may have enhanced imaging mass with periductal fat stranding under computed tomography and magnetic resonance imaging, and raised serum carbohydrate antigen 19-9, which can be misdiagnosed as cholangiocarcinoma. Contrast-enhanced ultrasound may be useful in diagnosis of traumatic neuroma. The imaging manifestations of a case of extrahepatic bile duct traumatic neuroma with contrast-enhanced ultrasound reported by Yuan show the enhanced mass, which is consistent with the enhanced mass shown by magnetic resonance imaging. They review images of 22 cases in 18 articles from 2000 to 2021. Ultrasonography features normally include hypoechoic or hyperechoic mass, local bile duct stricture, and proximal bile duct dilatation. Intrabiliary ultrasound can also play a role in its diagnosis. Intrabiliary ultrasound can accurately display lesional details, such as the presence of a homogeneous hypoechoic mass with clear borders, and reveal portal vein, hepatic artery, pancreas and presence of invasion.^[2]^ It suggests that it is superior in identifying the nature and origin of tumor.^[2]^ Mass biopsy is important for definitive diagnosis. A case ofbiliary neuroma is diagnosed by choledochoscopy biopsy in an asymptomatic patient, hence who avoids surgery.^[3]^ Two cases of traumatic neuroma post-cholecystectomy are diagnosed by a combined use of choledochoscopy and biopsy by intrabiliary ultrasound.^[3,4]^ Endoscopic retrograde cholangiopancreatography can provide both diagnostic and therapeutic options by visualizing bile duct and allowing for biopsy or stent placement.

At present, these diagnostic data come from case reports, and further research is still needed to verify their diagnostic value. An accurate diagnosis of biliary traumatic neuroma pre-surgery is difficult, so it is easy to be misdiagnosed. Therefore, it is necessary to combine the history of biliary tract surgery, clinical manifestations, imaging findings, laboratory tests and biopsy to rule out malignant tumors. Asymptomatic cases may be observed with images. Surgical resection of neuroma and reconstruction of bile duct are the mainstream treatment for symptomatic cases. When the neuroma is removed along with good blood supply and no tension of anastomotic edges of proximal and distal bile duct, end-to-end bile duct anastomosis can be performed. During surgery, it is important to differentiate it from malignancies, because the treatment approaches are different.^[5,6]^ Therefore, attention should be paid to frozen section pathology examination to exclude malignant tumors, thereby avoiding unnecessary extensive radical surgery and increasing the risk of postoperative complications and financial burden. Endoscopic interventions, such as stent placement, may be used to relieve obstruction temporarily or in patients who are not surgical 146 candidates. The prognosis is generally good after surgical treatment, but recurrence is possible if the underlying cause such as chronic irritation or trauma persists. Long-term follow-up is necessary to monitor complications such as recurrent obstruction or stricture.

Our case highlights the importance of careful surgical techniques during cholecystectomy to avoid bile duct injury and the potential for late complications such as traumatic neuroma. In cases where severe inflammation or fibrosis in Calot’s triangle, dissection of cystic duct is difficultly conducted, and subtotal cholecystectomy can be done, which is an easy, safe and definitive approach.^[7]^

## 8. Conclusions

This case highlights the importance of careful surgical technique during cholecystectomy to avoid bile duct injury and the potential for late complications such as traumatic neuroma. Frozen section pathology examination during surgery can help avoid unnecessary extensive radical surgery.

## Author contributions

**Data curation:** Zhao Chen.

**Supervision:** Tingting Zhu.

**Writing – original draft:** Guanbai Cao.

**Writing – review & editing:** Jun Wu.
